# Case report: Rare abernethy malformation with hepatopulmonary syndrome in a pediatric patient

**DOI:** 10.3389/fped.2022.856611

**Published:** 2022-09-09

**Authors:** Lianfu Ji, Zhaoming Ji, Dandan Xiang, Yuming Qin, Shiwei Yang

**Affiliations:** Department of Cardiology, Children's Hospital of Nanjing Medical University, Nanjing, China

**Keywords:** abernethy malformation, hepatic portal vein system, hepatopulmonary syndrome, splenic arteriography, inferior vena cava balloon occlusion test

## Abstract

Abernethy malformation is a rare abnormality of the hepatic portal vein system with non-specific and diverse clinical manifestations. Here, we described a case of abernethy malformation with hepatopulmonary syndrome in a 10-year-old girl. On physical examination, cyanosed lips and acropachy could be found. Her oxygen saturation fluctuated at 89–94%, and the fasting blood ammonia was 98 umol/L. Furthermore, there were abnormalities in the imaging. The microbubble test with contrast echocardiography was positive. Computer tomography angiography (CTA) showed the splenic vein, and the superior mesenteric drained directly into the inferior vena cave after confluence. The same result was also observed in delayed splenic arteriography. Then, we discovered a tiny branch of the intrahepatic portal vein by the inferior vena cava balloon occlusion test, which could also show the confluence of the splenic vein and superior mesenteric vein with the inferior venacave. According to the evidence above, we concluded that the girl was a patient of type II abernethy malformation. For the severe dysplasia of the portal vein, the girl accepted partial ligation of portosystemic shunt and Rex shunt, which improved her oxygen saturation and exercise tolerance.

## Introduction

Abernethy malformation, also known as congenital extrahepatic portosystemic shunts (1), is a rare abnormality of the hepatic portal vein system, with a common feature that the blood of the hepatic portal vein system shunts directly into the body vein system without passing through the liver. Variation of the vascular anatomical pathway and the different scopes and degrees of involvement may result in abnormal hemodynamics, which can cause many serious complications, such as hepatic encephalopathy (HE) (2), hepatopulmonary syndrome (HPS) (3), pulmonary arterial hypertension (HPA) (4), liver tumors (5), or glomerulonephritis (6). In recent years, with the gradual understanding of this disease, clinical case reports have increased. Here, we described a case of HPS secondary to type II abernethy malformation in a 10-year-old girl.

## Clinical presentation

**Figure 1:**
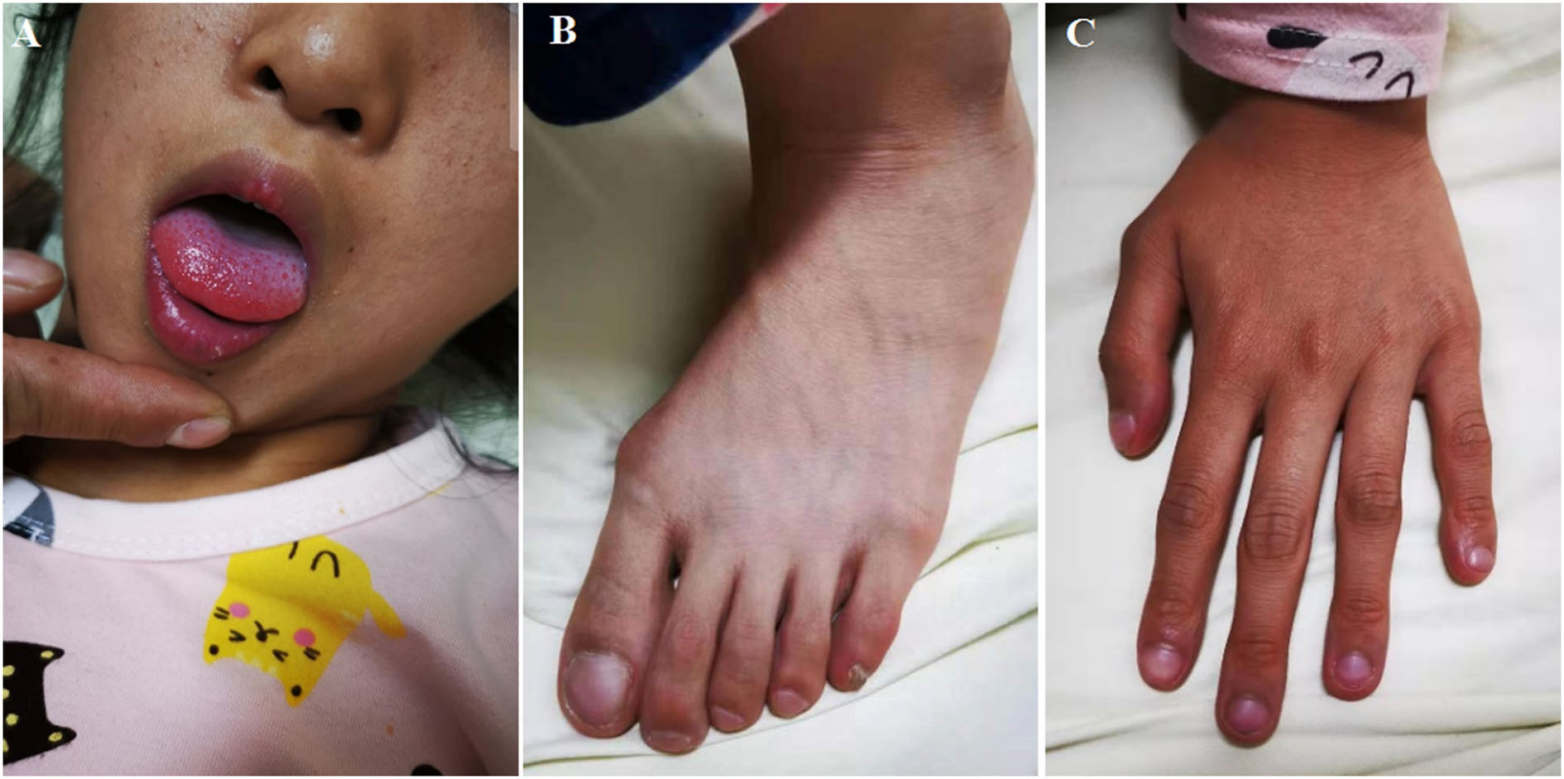
The girl has mild cyanosed lips **(A)**, digital clubbing in toes **(B)** and fingers **(C)** of high-density shadows in both lungs. Electrocardiogram (ECG) and echocardiogram were both normal.

A 10-year-old girl, who has suffered from persistent cough and slightly cyanosed lips for 5 years, was admitted to the cardiovascular department. in our hospital. The girl has weakness, fatigue, shortness of breath after activity, and she presented a history of recurrent respiratory infections (4–5 times a year). On physical examination, we found the girl had cyanosed lips and acropachy (see Figure 1). Her vital signs were as follows: the heart rate of 124/min; the respiratory rate of 25/min; blood pressure of 102/55 mmHg. The oxygen saturation fluctuated between 89–94%, while blood gas analysis showed PaO_2_51.5 mmHg and SpO_2_ of 85.6%. The fasting blood ammonia was elevated to 98 umol/L, and hemoglobin (HGB) was 155 g/L. Liver function and myocardial enzyme levels were normal. No obvious abnormality was found in lung function tests and brain MRI. Chest X-ray showed multiple small flocculent pieces.

**Figure 2:**
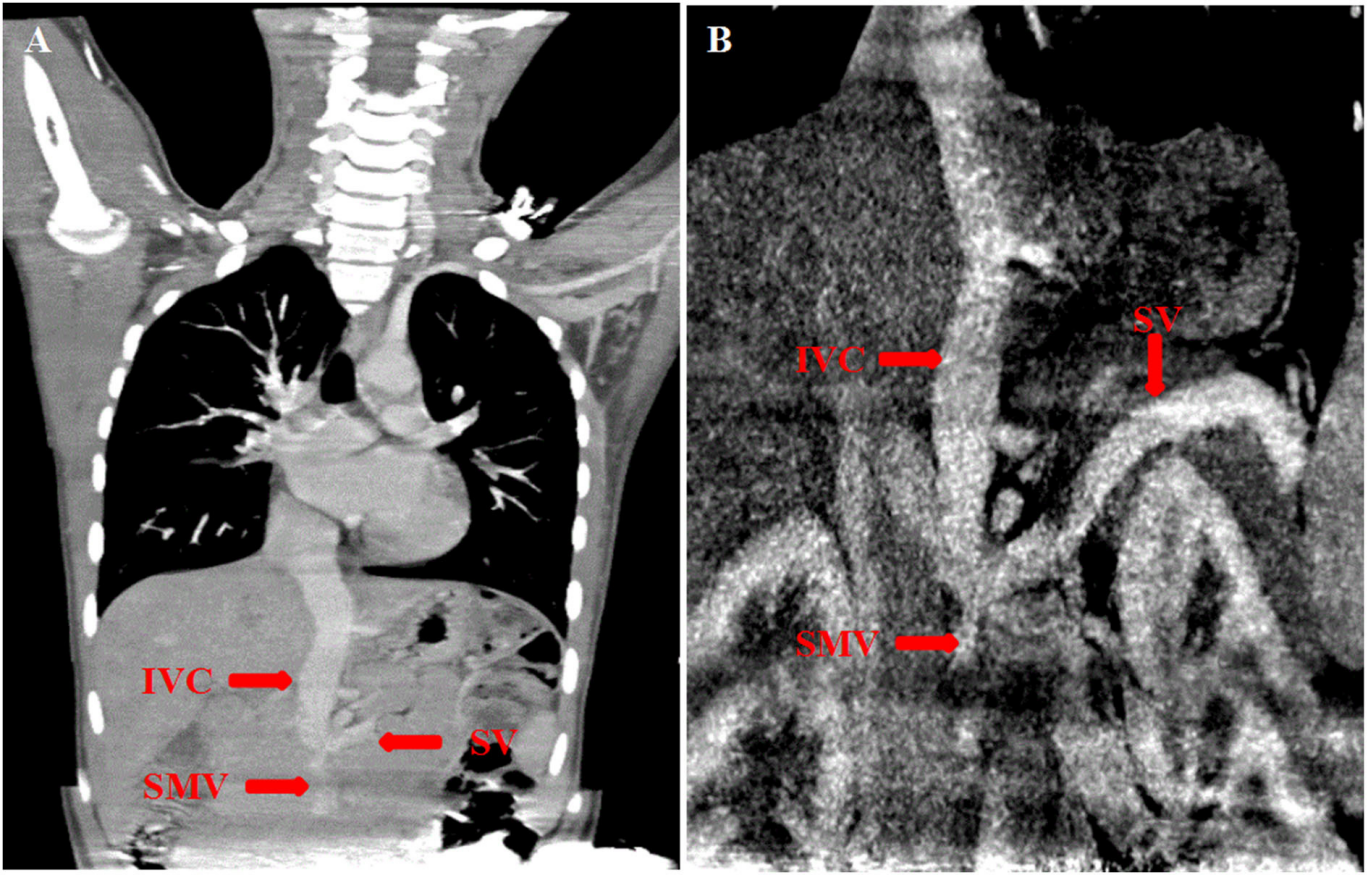
**(A,B)** Computer tomography angiography (CTA) showed the splenic vein (SV) joined the superior mesenteric vein (SMV) and then flowed upward into the inferior vena cava (IVC) directly.

Then, we performed the contrast echocardiography that showed microbubbles appeared in the left atrium after four cardiac cycles, reminding us pulmonary arteriovenous fistula should be taken into account. Next, CTA was recommended, which showed the splenic vein and the superior mesenteric drained into the inferior vena cava directly after confluence. We came to a conclusion that the girl was a patient of abernethy malformation (see Figure 2).

**Figure 3:**
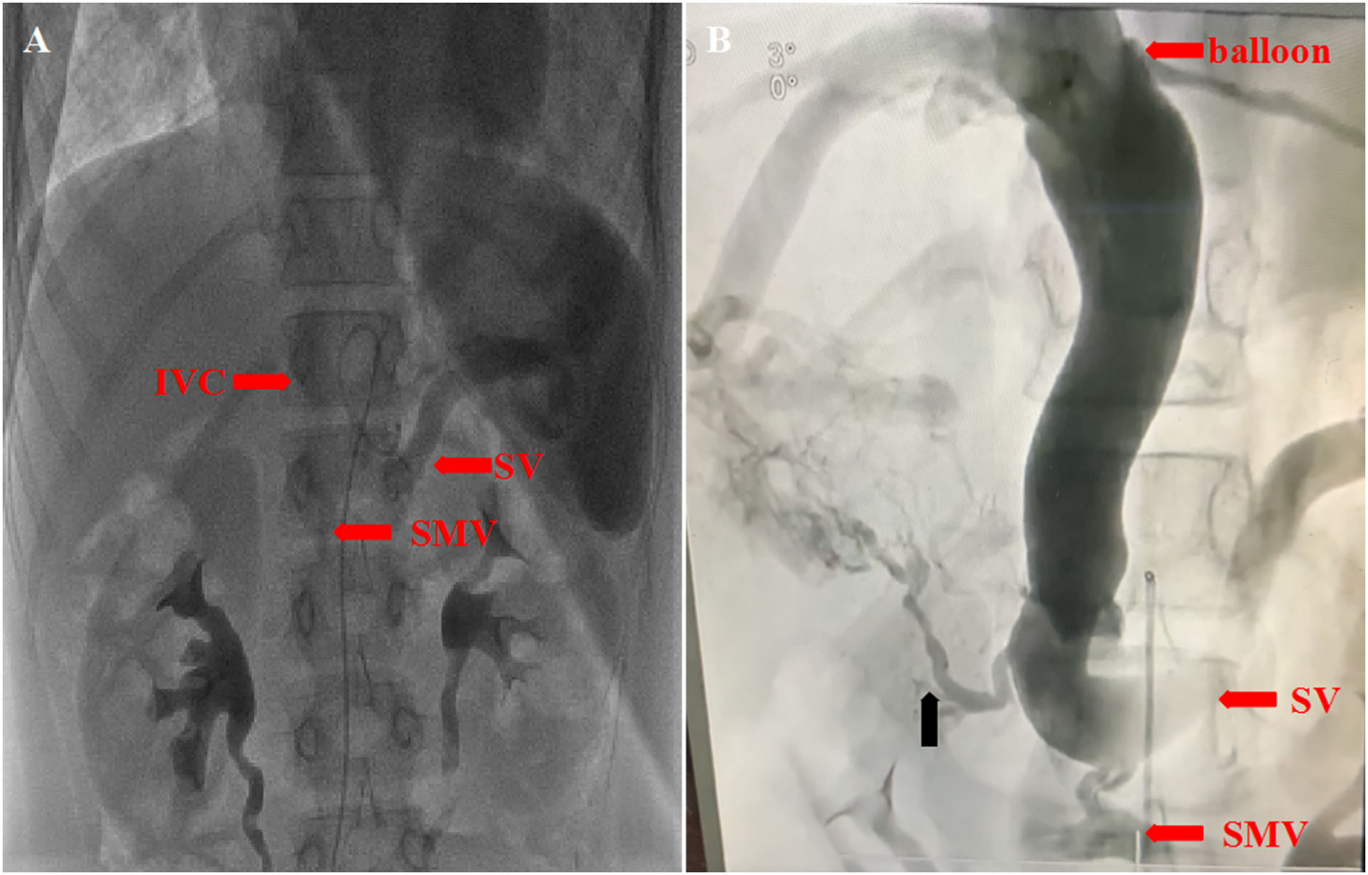
**(A)** Delayed splenic arteriography showed that the splenic vein (SV) joined the superior mesenteric vein (SMV), which merged into the inferior vena cava (IVC) directly. **(B)** The inferior vena cava balloon occlusion test showed a tiny branch of the intrahepatic portal vein (the black arrow).

To clarify the diagnosis further, we performed angiography. Right cardiac catheterization revealed that the pulmonary arterial pressure was within the normal range (24/16 mmHg). An ortography showed no abnormality in aorta and pulmonary artery traffic, and selective pulmonary angiography revealed no local pulmonary arteriovenous fistula. According to delayed splenic arteriography, the splenic vein joined the superior mesenteric vein, which merged into the inferior vena cava directly. We also performed the inferior vena cava balloon occlusion test, which can not only clearly show the confluence of the splenic vein and superior mesenteric vein with the inferior vena cava, but also helped us find a tiny branch of the intrahepatic portal vein (see Figure 3). Due to the severe dysplasia of the portal vein, the girl accepted partial ligation of the portosystemic shunt and the Rex shunt. After shunt closure, her exercise tolerance and oxygen saturation improved. We are having a close follow-up with the girl.

## Discussion

Abernethy malformation is classified into two types based on the abnormal shunt between the hepatic portal vein and the body vein: type I and type II. In type I, all blood from the splenic vein and the superior mesenteric vein drains into the body vein because there are no intrahepatic portal vein branches. Patients of type II have branches of the intrahepatic portal vein, which means part of the blood from the splenic vein and the superior mesenteric vein could drain back to the liver (7, 8). Liver transplantation is the only curative treatment for type I shunts, while patients of type II should be treated by different methods based on the development of intrahepatic portal vein branches (9). The therapeutic options for type II abernethy malformation include surgical ligation and transcatheter closure of the shunt (10, 11). With a deeper understanding, interventional radiology is the preferred method of closure (12). But it is up to the surgeon making a choice based on the patient.

The clinical manifestations of abernethy malformation are non-specific and diverse. Here, we reviewed the entire diagnostic process of the case in detail. Owing to the girl's signs of long-term hypoxia, we first considered various common heart and lung diseases, such as obstructive sleep apnea syndrome (OSA), bronchopulmonary dysplasia (BPD), Bronchiolitis obliterans (BO), congenital heart disease with right-to-left shunt, etc. To look for clues, we performed the relevant examinations, including lung function, X-ray of the nasopharynx and the chest, echocardiography, microbubble tests with contrast echocardiography, and computer tomography angiography (CTA). CTA revealed that the splenic vein joined the superior mesenteric vein and then flowed directly into the inferior vena cave. Chest X-ray showed multiple small flocculent pieces of high-density shadows in both lungs, and the microbubble test was positive. Based on the evidence mentioned above, the abernethy malformation with hepatopulmonary syndrome (HPS) should be taken into account. Vasoactive substances, including blood ammonia entering pulmonary circulation without being deactivated by the liver, cause the pulmonary capillaries dilated, which can trigger off pulmonary arteriovenous fifistula (13, 14).

Correct classification of abernethy malformation is critical to treatment selection. But it is difficult to find the tiny intrahepatic portal vein branch in type II cases with dysplasia of the portal vein branches by CTA and easily misdiagnose it as type I case. Angiography is a sensitive and accurate method for determining definitive diagnosis and classification of abernethy malformation. What is more, we need to use different angiography methods to determine the presence and development of intrahepatic portal vein branches, including superior mesenteric vein angiography, the delayed superior mesenteric artery angiography or portal venography after temporary shunt occlusion by balloon, etc. In this case, a tiny branch of the intrahepatic portal vein was revealed by the inferior vena cava balloon occlusion test. So the diagnosis of type II abernethy malformation was clear.

In summary, as clinicians, abernethy malformation should be taken into account when the children suffered from unexplained cyanosis. It should be stressed that correct classification of abernethy malformation is critical to treatment selection.

## Data availability statement

The original contributions presented in the study are included in the article/supplementary material, further inquiries can be directed to the corresponding author/s.

## Ethics statement

The studies involving human participants were reviewed and approved by the Ethics Committee of the Children's Hospital Affiliated to Nanjing Medical University. Written informed consent to participate in this case study was provided by the participants' legal guardian/next of kin. Written informed consent was obtained from the individual(s), and minor(s)' legal guardian/next of kin, for the publication of any potentially identifiable images or data included in this article.

## Author contributions

LJ and ZJ edited the manuscript. DX contributed samples collection. YQ and SY revised the paper. All authors contributed to the article and approved the submitted version.

## Funding

This work was supported by Nanjing Medical Science and Technology Development Fund (YKK19109/ZKX20041/QRX17024), Medical Research Project of Jiangsu Commission of Health (ZD2021058), and Jiangsu Maternal and Child Health Research Project (F202023).

## Conflict of interest

The authors declare that the research was conducted in the absence of any commercial or financial relationships that could be construed as a potential conflict of interest.

## Publisher's note

All claims expressed in this article are solely those of the authors and do not necessarily represent those of their affiliated organizations, or those of the publisher, the editors and the reviewers. Any product that may be evaluated in this article, or claim that may be made by its manufacturer, is not guaranteed or endorsed by the publisher.
